# Sulforaphane and Other Nutrigenomic Nrf2 Activators: Can the Clinician's Expectation Be Matched by the Reality?

**DOI:** 10.1155/2016/7857186

**Published:** 2016-01-06

**Authors:** Christine A. Houghton, Robert G. Fassett, Jeff S. Coombes

**Affiliations:** School of Human Movement and Nutrition Science, The University of Queensland, Brisbane, Australia

## Abstract

The recognition that food-derived nonnutrient molecules can modulate gene expression to influence intracellular molecular mechanisms has seen the emergence of the fields of nutrigenomics and nutrigenetics. The aim of this review is to describe the properties of nutrigenomic activators of transcription factor Nrf2 (nuclear factor erythroid 2-related factor 2), comparing the potential for sulforaphane and other phytochemicals to demonstrate clinical efficacy as complementary medicines. Broccoli-derived sulforaphane emerges as a phytochemical with this capability, with oral doses capable of favourably modifying genes associated with chemoprevention. Compared with widely used phytochemical-based supplements like curcumin, silymarin, and resveratrol, sulforaphane more potently activates Nrf2 to induce the expression of a battery of cytoprotective genes. By virtue of its lipophilic nature and low molecular weight, sulforaphane displays significantly higher bioavailability than the polyphenol-based dietary supplements that also activate Nrf2. Nrf2 activation induces cytoprotective genes such as those playing key roles in cellular defense mechanisms including redox status and detoxification. Both its high bioavailability and significant Nrf2 inducer capacity contribute to the therapeutic potential of sulforaphane-yielding supplements.

## 1. Introduction

Whilst early 20th century nutrition science resolved issues related to micronutrient deficiency states and the latter part focused more on macronutrient excesses [1], the first decade of the 21st century has already seen old paradigms challenged and new theories proposed. The recognition that food-derived nonnutrient molecules can modulate intracellular molecular mechanisms has seen the emergence of the fields of nutrigenomics and nutrigenetics, disciplines derived from the interweaving of the sciences of nutrition, biochemistry, molecular biology, and genomics. It has been estimated that there are more than 5000 different phytochemicals present in food [2] and our current knowledge is limited to a reasonable understanding of the function of just a few.

Against this background sits the quest to identify biomolecules with significant nutrigenomic potential. A growing body of research highlights one such biomolecule, sulforaphane, an isothiocyanate (ITC) derived from the cruciferous vegetable family and in particular from* Brassica oleracea* [3]. Although the plant kingdom is the source of thousands of phytochemicals, little is known about the way in which food-derived phytochemicals support the maintenance of human health and especially those associated with cellular defense mechanisms. As the science of nutrigenomics evolves and our understanding of the many interactions between phytochemicals and endogenous cytoprotective mechanisms grows, the significance of plant foods in human health becomes clearer.

A critical review of the formulations of some available supplements reveals numerous flaws, shedding doubt on their potential efficacy [4]. There are few published clinical trials using phytochemicals as the intervention material and only a small number of these withstand scientific scrutiny. However, even when benefit for a compound has been demonstrated, it is common for a commercial product to include the ingredient at a dose manyfold lower than that shown to be efficacious in either clinical trials or as it was traditionally employed by cultures of the past. As a further trap for the unwary consumer or uninformed clinician, supporting commentary may include citations for in vitro and animal studies, giving the reader a false impression of the product's likely efficacy as a supplement for humans.

Because it appears that many consumers have accepted a role for complementary medicines in their personal health management, it is important to review the evidence on whether plant-derived supplements can assist in modifying various biochemical and physiological risk factors for disease. The aim of this review is to describe the properties of nutrigenomic activators of Nrf2, focusing on the potential for sulforaphane and other activators of gene expression to demonstrate clinical efficacy as complementary medicines.

## 2. Beyond Nutritional Deficiencies and Excesses

### 2.1. Nutrigenetics and Nutrigenomics

The interlinked sciences of nutrigenetics and nutrigenomics provide the clinician with a more targeted opportunity to personalise a patient's treatment programme [5], revealing those genetic polymorphisms which may compromise individual biochemical function. Even without access to sophisticated genome profiling, a clinician's knowledge that potent food-derived biomolecules can interact with intracellular signaling pathways provides another dimension to clinical management and disease prevention processes.

The realization that food-derived molecules are in constant conversation with complex intracellular control systems via signaling pathways has unveiled the role of food as so much more than a source of micro- and macronutrients [6]. What becomes immediately apparent in this model is that no multinutrient supplement can substitute for the enormous diversity in phytochemicals present in a balanced human diet. Also evident is that the health benefits of the popular polyphenolic phytochemicals such as those found in green tea, grape seed, red wine, curcumin, pomegranate, and olives are unlikely to be due to direct-acting antioxidant effects demonstrated by these molecules in numerous in vitro studies [7, 8]. Polyphenols are typically large bulky molecules which are poorly absorbed and poorly bioavailable [9], so that it is unlikely that the intracellular micromolar concentrations necessary to scavenge free radicals can be achieved. Polyphenols can also behave as either antioxidants or prooxidants depending on the experimental conditions [10]. In addition, newer evidence suggests polyphenols and other phytochemicals may function hormetically, whereby dose response is characterised by low dose stimulatory response and high dose inhibition [11].

In a bioactive-specific approach, a recent comprehensive review of phytochemicals indicated for cardiovascular disease focused on both preclinical and clinical beneficial effects of four commonly supplemented compounds [12]. The review concluded that there are few definitive trials in this area and in some studies the exact dose used is not clear. However, the authors confirm the findings of others in that the use of a very high dose is associated with the most protective effects for a few phytochemicals, whereas the lowest dose turns out to be the most effective for other compounds.

As with vitamin “antioxidants,” the notion that ingested polyphenol supplements act as “antioxidants” in human cells is called into question [7]. Emerging evidence suggests that polyphenols or their metabolites exert their systemic intracellular effects not as direct “antioxidants” per se but as modulators of signaling pathways.

### 2.2. Cruciferous Vegetables Harbor Nutrigenomic Potential

The classification, cruciferous vegetables (crucifers), includes species predominantly from Brassicaceae family and the more common members are cultivars not only of* Brassica oleracea* genus including broccoli, cabbage, cauliflower, Brussels' sprout, and kale but also of* Raphanus* genus which includes various types of radish. Although these vegetables are good sources of micronutrients, their value to human health would seem to be at least partly due to the nature of the phytochemicals they contain and in particular the glucosinolates [13], the enzymatic hydrolysis products of which are capable of modifying gene expression [14]. Although vegetables such as broccoli are not popular dietary choices [15], the unique health-promoting value of crucifers continues to be reaffirmed [16]. A recent review [17] investigating the effect of crucifers on total and cardiovascular mortality found that several prospective studies showed no association for total vegetable consumption but did show a significant inverse association for cruciferous vegetable consumption. The potential benefits of green leafy vegetables in general and cruciferous vegetables in particular are not limited to their effects in cancer and cardiovascular disease. In a 27-year prospective cohort study on cognitive decline in ageing women (*n* = 15,080), those in the highest quintile of cruciferous vegetable intake declined more slowly than those in the lowest quintile, with an evident linear dose response [18]. Those in the highest quintile of green leafy vegetable intake also experienced slower cognitive decline. The association did not change when data for participants with cardiovascular disease and diabetes were excluded.

Most research on crucifers has focused on broccoli,* Brassica oleracea* (both vegetable and sprouts), as a source of bioactive compounds with nutrigenomic potential. The last two decades have seen accelerating interest in the role of broccoli in human health following evidence that induction of detoxification enzymes might be responsible for the majority of the observed health benefits of vegetables [19, 20]. After isolating broccoli-derived sulforaphane, Zhang's group showed that sulforaphane was a major and very potent Phase II enzyme inducer. The group of induced enzymes includes NAD(P)H:NQO1 (quinone reductase) and the family of glutathione-S-transferases (GSTs), both of which are required for the detoxification of steroids and the ubiquitous environmental toxin, benzo(a)pyrene [21–23]. Zhang et al. concluded that the induction of detoxification enzymes by sulforaphane may significantly contribute to the anticarcinogenic action of broccoli. The way that sulforaphane demonstrably increased target enzymes is indicative of a nutrigenomic effect, even though the precise mechanism to explain such gene expression was not known at the time. It would be another two years before the mechanism to explain the effect of sulforaphane would be elucidated [24].

## 3. Influencing Signaling Pathways

### 3.1. Nrf2 as “Master Regulator” of Cell Defense

Although sulforaphane interacts in a number of mammalian biochemical pathways, its effect on the redox-sensitive transcription factor, Nrf2 (nuclear factor erythroid 2-related factor 2), appears to be responsible for its greatest clinical potential when administered at practical oral doses [25]. Reference to Nrf2 first appeared in the scientific literature in 1994 and has subsequently been the subject of over 5,500 MEDLINE published papers [24]. In the ensuing two decades, Nrf2 has emerged as a key modulator of the cell's primary defense mechanism, countering many harmful environmental toxicants and carcinogens [26]. Considerable research has focused on Nrf2's role in preventing the activation of carcinogens to toxic metabolites, especially by induction of the Phase II detoxification enzyme, NAD(P)H:quinone reductase (NQO1) [27].

The elucidation of the mechanism by which Nrf2 acts as a cytoplasmic “switch” to activate a battery of cytoprotective genes arguably heralds a new paradigm in nutrition science. Identification of Nrf2 gave the first real clue that bioactive diet-derived compounds like sulforaphane had the potential to coordinately influence large banks of function-specific genes [28].

Nrf2 has been variously described as an activator of cellular defense mechanisms [29], the master redox switch [30] and a guardian of health span and gatekeeper of species longevity [31]. As a mediator for amplification of the mammalian defense system against various stressors, Nrf2 sits at the interface between our prior understanding of oxidative stress and the endogenous mechanisms cells use to deal with it. What has become clear is that although attempts to counter oxidative stress by “antioxidant” vitamin supplementation have been disappointing [32], many phytochemicals have the capacity to activate Nrf2 and thereby induce genes [33] which collectively regulate much of the cell's endogenous defense system, enhancing its survival [34]. This finding may be clinically significant in that diseases known to be underpinned by oxidative stress may prove to be more responsive to such amplification of cellular defenses via Nrf2 activation compared to by the administration of direct-acting antioxidant supplements [35].

### 3.2. Sulforaphane, an Inducer of Nrf2 Target Genes

Notably and perhaps surprisingly, given its significant cytoprotective potential, sulforaphane does not exhibit a direct antioxidant effect; instead it is weakly prooxidant [36]. As further evidence to support the critical role of redox signaling in cellular defense mechanisms, the ability of sulforaphane to induce NQO1 and cell cycle arrest in prostate cancer cell lines was shown to have been completely abrogated by pretreatment with the glutathione (GSH) precursor, N-acetylcysteine [37, 38]. This finding has implications for the regular ingestion of readily available supplements of N-acetylcysteine.

Sulforaphane [1-isothiocyanato-(4R)-(methylsulfinyl) butane: CH_3_S(O)(CH_2_)_4_-N=C=S] is a small (MW = 177.29) aliphatic lipophilic organosulfur molecule which is not present in cruciferous or other plants (Supplementary Data, Figure 1, in Supplementary Material available online at http://dx.doi.org/10.1155/2016/7857186). Instead, plants of* Brassica* genus contain a biologically inactive precursor compound, glucoraphanin (GRN), which is contained within a plant cell vacuole together with an enzyme, myrosinase (MYR), which is separately compartmentalized [39]. It is when the plant cell ruptures and the GRN and MYR come into contact that sulforaphane is enzymatically produced [40] (Supplementary Data, Figure 2). Compared with its stable GRN precursor, the resulting sulforaphane aglycone is relatively unstable [41]; this has implications for culinary applications of broccoli and other cruciferous vegetables. Broccoli is not the only crucifer which yields sulforaphane but it yields the highest amounts, with its GRN content around 75% [42] of total glucosinolates. Notably, glucosinolate-containing plants contain variable quantities of both precursor and enzyme [43]. As a result, the yield of sulforaphane and other isothiocyanates can vary widely.

Cutting, chewing, or otherwise disrupting the broccoli plant cell structure initiates the synthesis of sulforaphane which, compared to its stable GRN precursor, begins degrading soon after synthesis [44]. For consumers to take advantage of the cytoprotective benefits of broccoli and other crucifers, steps must be taken to conserve the integrity of the sulforaphane released.

Sulforaphane belongs to one of nine identified classes of chemical Nrf2 activator [45]. Structurally varied, the only property shared by all inducers is their ability to react with sulfhydryl (-SH) groups. Nrf2 therefore is intimately tied to sulfur chemistry and provided dietary protein is adequate, a balanced diet should furnish sufficient sulfur. However, there are concerns that sulfur intake in many may be marginal [46], with some researchers suggesting that deficiency of sulfur amino acids can compromise GSH synthesis to a greater extent than for protein synthesis in both the presence and absence of inflammatory stimuli [47]. Whilst vegan diets may provide significant levels of phytochemicals [48], there may be a need for vigilance regarding sulfur adequacy, given that the sulfur-containing amino acids are least abundant in plant proteins and that vegans typically consume about half of the sulfur consumed by those consuming a mixed balanced diet [46].

### 3.3. Broccoli Sprout versus Broccoli Vegetable

Much of the clinically relevant* Brassica* research relates to broccoli sprouts [49] rather than to the mature vegetable, with most of the early work in this field done by a group at the Johns Hopkins University beginning in the early 1990s. The group found that 3-day-old sprouts of cultivars of certain crucifers contained 10–100 times higher concentration of GRN than the corresponding mature plants [49]. With a focus on identifying plants with cancer chemopreventive properties, they found that the sprouts were highly effective in reducing the incidence, multiplicity, and rate of development of mammary tumors in dimethylbenz(a)anthracene-treated rats. Broccoli sprouts also had the added advantage of containing mostly the methylsulfinylalkyl glucosinolate (75% of the total) and very little of the indole glucosinolate found in the mature plant, which is a potential tumor promoter [50]. Their realization was that small quantities of broccoli sprouts may protect against cancer as effectively as much larger quantities of the vegetable stimulated subsequent research.

### 3.4. How Nrf2 Activators Influence Gene Expression

**Figure 1:**
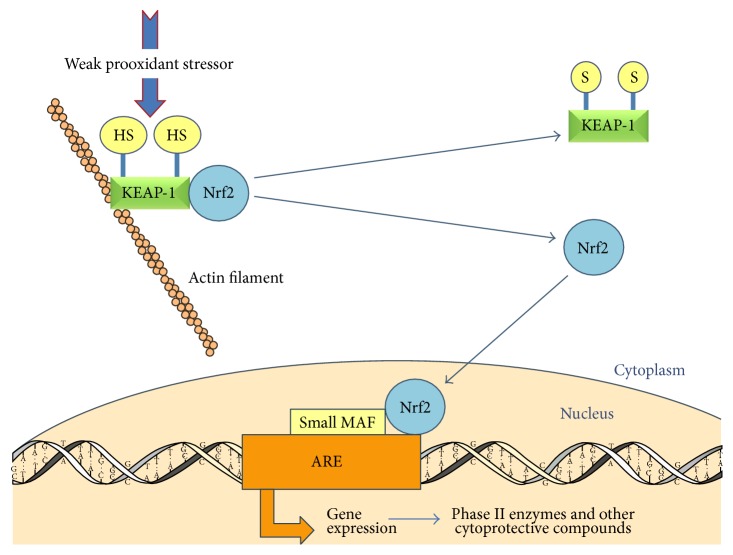
The mechanism by which Nrf2 activation increases the expression of genes with an Antioxidant Response Element (ARE) in their promoter regions. Human Keap-1 contains 27 cysteine residues providing sulfhydryl groups (-SH) which act as sensors of ARE inducers including oxidative stress [69]. Small Maf protein is essential for Nrf2 function [170]. Figure adapted from Kensler et al., 2003, with permission [51].

Although the complexity of Nrf2 pathways has not yet been fully elucidated, the principal elements are depicted in Figure 1 [51]. Essentially, Nrf2 is sequestered in the cytoplasm by the actin-bound cytosolic repressor Keap-1 (Kelch-like ECH-associated protein 1), a cysteine-rich protein which also acts as a sensor of variations in cytoplasmic redox status. When the appropriate signal is detected by cysteine thiols within Keap-1, its ability to bind and retain the transcription factor Nrf2 in the cytoplasm is lost. Keap-1 typically responds to an electrophilic or oxidative stress signal [51].

Thus released, Nrf2 translocates to the nucleus where it aligns with a short nucleotide base sequence in the promoter region of its target genes; this sequence is commonly known as the Antioxidant Response Element (ARE) or the Electrophilic Response Element (EpRE), with the latter being considered a more correct descriptor, although the terms are used interchangeably [52]. To bind, Nrf2 dimerizes with other basic leucine zipper (bZIP) proteins such as small Maf proteins (Maf G) to form a transactivation complex that binds to AREs [53].

When an electrophilic or oxidative stressor challenges the cell, Keap-1 senses the disturbance to its cytoplasmic redox equilibrium. After release from Keap-1, Nrf2 levels rapidly rise in the nucleus, upregulating a battery of cytoprotective genes, each containing at least one ARE. Of significance is the effect of Nrf2 on induction of the rate-limiting enzyme for (GSH) synthesis, *γ*-glutamyl-cysteine synthetase, thereby elevating tissue GSH levels [54].

For the Nrf2-Keap-1 pathway to such a play a key role in cytoprotection, its activity must be capable of being regulated in tandem with the ever-changing cellular environment. Under basal nonstressed conditions, Nrf2 is continuously degraded via the ubiquitin-proteasome pathway [55]. With a half-life of around 20 minutes [56], Nrf2 is maintained at a low cellular level [57]. Exposure to stressors inactivates Keap-1 by direct modification of cysteine thiol residues, thereafter releasing Nrf2 in a derepression-type stress response [58].

The clinical significance of this mechanism is apparent when considering the hepatotoxic effects of acetaminophen, a drug responsible for considerable drug-induced liver injury [59]. Excessive doses of this common analgesic/antipyretic drug rapidly deplete intracellular GSH reserves. However, the cell activates an adaptive response whereby Keap-1 senses the acetaminophen metabolite, N-acetyl-p-benzoquinone imine (NAPQI), subsequently activating Nrf2 [60]. GSH is synthesised rapidly along with a battery of other Nrf2 target genes. This mechanism may not be adequate to increase GSH levels in an acute care setting, given that translation times for protein synthesis of various Nrf2 target genes can take hours. A study investigating the effect on gene expression of cytoprotective heme oxygenase-1 (HO-1) in neurons after subarachnoid haemorrhage showed that Nrf2 levels increased ~4-fold at 12 hours, peaking at >4.5-fold at 24 hours, with HO-1 levels increased to >3-fold at 12 hours and peaking at >4.5-fold at 24 hours [61].

### 3.5. Phase II Enzymes and the Detoxification Mechanisms

The mechanisms that cells use to detoxify potentially harmful compounds, often carcinogens [62, 63], can involve a Phase I component associated with monoamine oxidases of the Cytochrome P450 family and a Phase II component where the intermediate compound produced by Phase I is metabolised in a way that permits ready excretion. A compound which activates Phase I and Phase II enzymes is known as a bifunctional inducer; however, if it activates only Phase II enzymes, it is a monofunctional inducer [64]. Phase II enzymes are induced by Nrf2 and as such are integral to this discussion. For safe and efficient detoxification, a toxin will ideally undergo a relatively slow Phase I reaction followed by a more rapid Phase II; this tends to prevent accumulation of the Phase I metabolite which can be more toxic than its precursor [65].

Therefore, for an optimal cellular detoxification environment, Phase II reactions should be at a rate which prevents intermediate products of Phase I from accumulating. Aliphatic sulforaphane acts as a monofunctional inducer, whereas the indole ITCs from mature broccoli are bifunctional inducers derived from the glucosinolate, glucobrassicin [49]. Of clinical significance is the finding that Phase II enzymes have a relatively long half-life, so that upregulated expression of these proteins can remain for several days. In a study using human adult retinal pigment epithelial cells (ARPE-19), NAD(P)H:quinone reductase remained active for more than 5 days [66].

## 4. Inducers of Nrf2 Target Genes

Nrf2 can be activated by a variety of inducers, not all of which are obtained orally. For example, the prooxidant signals generated by the reactive oxygen species released during exercise [67] or from inhaled environmental chemicals [68] are capable of upregulating the cellular endogenous defenses, provided exposure is sufficiently modest that it does not overwhelm the cell's defenses.

### 4.1. Diet-Derived Nrf2 Inducers

Although a number of phytochemicals have been investigated in relation to their Nrf2 inducer ability, the mechanistic studies to explain the nature of the induction are limited. A review paper focused on molecular mechanisms of phytochemicals in chemoprevention suggested that only three plant-derived molecules, sulforaphane, carnosol, and quercetin, have been mechanistically investigated in this regard and only sulforaphane has been studied for its roles in multiple mechanisms [69]. Given the more extensive literature on sulforaphane, we hereafter consider its potential as a supplement of clinical significance and where the data exist, comparing its potential with that of popular and widely available phytochemical supplements.

### 4.2. Sulforaphane: In Vitro Effects

Sulforaphane is a potent Nrf2 inducer with consequent induction of cellular defenses [70]. The effect is rapid in cell culture with activation by sulforaphane occurring within 30 minutes in human bronchial epithelial BEAS-2B cells [71]. Using microarray analysis to investigate the effect of sulforaphane in the wild-type murine liver, Hu et al. showed that expression levels of 1725 genes were increased after 3-hour exposure and 3396 genes were changed after 12 hours [33]. Comparing expression patterns at different time points, they also showed that maximal change occurred 12 hours after a single administration of sulforaphane, based on fold changes greater than 2-fold. The identified Nrf2 target genes can be classified broadly as those coding for a range of cytoprotective proteins, including antioxidants (enzyme and nonenzyme), drug-metabolising enzymes, drug-efflux pumps, heat shock proteins, NADPH regenerative enzymes, growth factors and growth factor receptors, heavy metal binding proteins, and various nuclear receptors including PPAR-*γ*, as well as for Nrf2 itself [33].

Vitamin D's protective effects on human cells are well recognized [72]; it may be nutritionally significant that the vitamin D receptor (VDR) is Nrf2 target gene inducible by sulforaphane [73]; in turn, Vitamin D can increase Nrf2 expression [74]. To further illustrate this diversity, Nrf2 target genes include those coding for *β*-defensin-2 (HBD-2), an antimicrobial peptide associated with innate immunity, protecting the intestinal mucosa against bacterial invasion. HBD-2 can be induced by sulforaphane [73] and was shown in a cell culture study using human Caco-2 cells to be significantly induced 1.6-fold at 24 hours and 2-fold at 48 hours by sulforaphane concentrations of >5 *μ*M. These results may have relevance in disorders of the intestinal epithelium but systemically an intracellular concentration of 5 *μ*M is probably higher than what can be readily achieved by diet or even via practical doses of available oral sulforaphane-yielding supplements.

The downstream enzyme products of Nrf2 target genes are efficient and versatile. They include those which constitute the glutathione and thioredoxin systems, the major cellular reducing systems in the body [75]. Several reasons explain their efficiency and versatility [76]: (1) they are not consumed stoichiometrically, as are direct-acting antioxidants such as ascorbate and tocopherols; (2) their duration of action is long with half-lives measured in days, so their induction need not be continuous; (3) they restore the endogenously produced direct-acting antioxidants like coenzyme Q10 and the tocopherols by returning them to the reduced state (in particular via NQO1 because both coenzyme Q10 and tocopherols are quinones). Major products of Nrf2 target genes and their roles in cytoprotection are listed in Supplementary Data, Table 1.

## 5. Quinone Reductase (NQO1), a Tool to Evaluate Inducer Capacity

Initially considered as an Nrf2-activated Phase II enzyme associated with detoxification pathways, the function of NQO1 is now considered to be much broader [77]. NQO1 has been described as a “quintessential cytoprotective enzyme” and is coded by what is considered “one of the most consistently and robustly inducible genes within its class” [77]. Furthermore, its activity declines with age whilst upregulation of its activity by Nrf2 induction is described as an avenue for maintaining cellular defenses with advancing age [31]. Furthermore, animal studies show significant decline in Nrf2 activity between youth and old age [78–80]. Humans genetically deficient in NQO1 are more susceptible to the carcinogenicity of benzene exposure [81]. NQO1 is highly active in pulmonary tissues [82] as well as in epithelial and endothelial cells in general [25], suggesting that it could act defensively against compounds absorbed via the airways, gut, and bloodstream. NQO1 activity is used as a rapid screening procedure and a biomarker of the anticarcinogenic activity of phytochemicals [45, 83]. The assay [20] uses cells defective in Phase I function to provide the means for selectively distinguishing monofunctional inducers that elevate Phase II enzymes [84].

### 5.1. CD Values as a Comparative Marker

The term “CD value” describes the concentration required to double NQO1 activity in murine hepatoma cells [85]. A CD value is also useful for comparing the potential in vivo nutrigenomic effects of an ingestible bioactive compound. The CD value has also been used [19, 83] to classify* Brassica* spp. according to their relative “anticancer potential.” When several crucifers were compared for their Nrf2 inducer effect [86], ITCs of cabbage, kale, and turnips exhibited less NQO1 inducer capacity than broccoli-derived sulforaphane. Sulforaphane returned ~33,000 units NQO1 inducer activity/g fresh weight for broccoli, cabbage returned ~11,000 units, and kale returned ~10,000 units with turnip returning ~2,000 units. This property may partly explain why broccoli is researched more extensively than other* Brassica* spp.

### 5.2. Clinical Significance of CD Value

**Figure 2:**
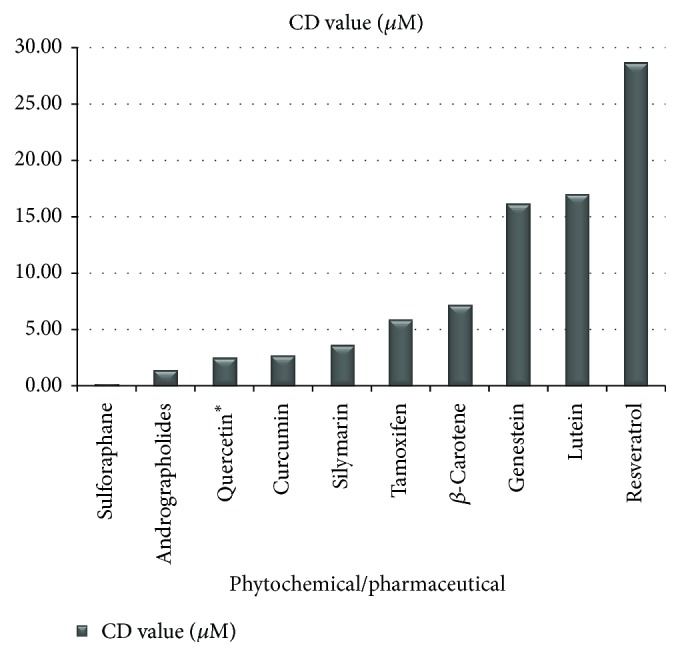
CD values of popular phytochemicals used as supplements and a commonly prescribed pharmaceutical. CD values refer to the concentration of a compound required to double the activity of the Phase II detoxification enzyme, quinone reductase [83, 87–89, 91].

In data from studies comparing CD values of well-known phytochemicals, sulforaphane showed the highest potency, with a concentration as low as 0.2 *μ*M required to double the activity of NQO1 [19, 85]. The comparative CD values of other phytochemicals have been documented by others [87–90], with lower micromolar concentrations representing those with the higher inducer activity (Figure 2).

CD values are available for phytochemicals used in common oral supplements [83, 87–89, 91]: sulforaphane (0.2 *μ*M), andrographolides (1.43), quercetin (2.50), *β*-carotene (7.2 *μ*M), lutein (*μ*M), resveratrol (21 *μ*M), indole-3-carbinol from mature broccoli vegetable (50 *μ*M), chlorophyll (250 *μ*M), *α*-cryptoxanthin (1.8 mM), and zeaxanthin (2.2 mM). An earlier study conducted in a different laboratory [91] had shown curcumin (2.7 *μ*M), silymarin (3.6 *μ*M), tamoxifen (5.9 *μ*M), genistein (16.2 *μ*M), epigallocatechin-3-gallate (EGCG) (>50 *μ*M), and ascorbic acid (>50 *μ*M). The comparative NQO1 inducer activity of these phytochemicals is sulforaphane > andrographolides > quercetin > curcumin > silymarin > tamoxifen > beta-carotene > genistein > lutein > resveratrol > I-3-C > chlorophyll > *α*-cryptoxanthin > zeaxanthin.

Notably, the CD value of sulforaphane is 13.5-fold greater than that of curcumin, 18-fold greater than silymarin, and 105-fold greater than resveratrol, all phytochemicals which are extensively promoted for their claimed health-promoting properties. It may be useful for relevant oral supplements to be evaluated in relation to the CD value of their primary ingredient(s), given that an Internet search will readily reveal many self-select and clinician-recommended supplements claiming to “enhance detoxification” and “promote longevity,” even though supporting evidence is not apparent. Many such supplements claiming to target “detox” are based on ingredients such as chlorophyll and vitamin C, both of which have comparatively low NQO1 inducer capacity.

### 5.3. Comparing Effects of Indole Glucosinolates

Indole-3-carbinol (I-3-C), the ITC found in mature broccoli vegetable (but not significantly in the sprout), required >50 *μ*M to double NQO1 activity [83]. In vivo, I-3-C must be dimerized in the acidic environment of the stomach to 3,3′-diindolymethane (DIM) to be active [92]. This has certain clinical implications as synthetic molecules of both I-3-C and DIM are available as supplements. With significantly lower inducer capacity than sulforaphane [91], it bears mention that DIM is also a bifunctional inducer of the detoxification pathway, thus limiting its cytoprotective potential. Early research on broccoli sprouts suggested potential limitations to the use of indole glucosinolates such as I-3-C as chemoprotectors in humans [49]. Not only are they weak inducers of Phase II enzymes but also, as bifunctional inducers, they simultaneously activate Phase I enzymes. They may also have estrogen receptor binding activity, adding to their potential as tumor promoters [49].

Interestingly, DIM is sometimes recommended clinically for patients with compromised estrogen metabolism, the theory being that DIM inhibits CYP1B. Inhibition of CYP1B1 shifts estrogen metabolism towards 4-hydroxyestrone, a metabolite which can contribute to carcinogenesis [93]. Not all data agree; a 2007 cell culture study analyzed gene expression using microarray profiling and quantitative real time-polymerase chain reaction in MCF7 breast cancer cells treated simultaneously with estradiol and DIM [94]. CYP1B1 was upregulated with a fold change of 3.93 ± 0.25. Such findings would tend to suggest that DIM may not protect against the metabolism of estrogen to the 4-hydroxy metabolites. Such conflicting data indicate that clinical trials are required to establish the in vivo effects of such an intervention when using a clinically relevant dose of a readily available supplement.

To illustrate the differences in potency between sulforaphane and I-3-C in a study using a prostate cell line, it was found that both compounds inhibited the proliferation of the prostate cancer cells in a dose-dependent manner but the inhibitory concentration of sulforaphane required was just 10% that of I-3-C [95]. There may also be safety issues which require caution in the recommendation of I-3-C supplements, available at many times the quantity of I-3-C achievable from broccoli vegetable consumption. Although I-3-C administered one week after the last dose of the carcinogen has been shown in rats to result in a latency delay of mammary tumor formation, it did not alter tumor incidence or multiplicity among survivors [96]. Any research showing a preventive benefit of this compound must be considered against the risk that it may promote liver and colon cancer [96].

### 5.4. Other Modes of Activating Nrf2

Although our focus is to compare the inducer capacity of phytochemicals, Nrf2 in human cells is activated by a range of stressors, not all of which are chemical in nature. The diverse nature of Nrf2 activators is highlighted in the three examples which follow. We use several examples of pharmaceuticals with pleiotropic Nrf2-inducing effects. Furthermore, we illustrate that when pharmaceutical Nrf2 activation occurs at supraphysiological levels, the outcome may be unexpected, indicating that the significantly lower inducer capacity of diet-derived Nrf2 activators may represent a hormetic effect [97].

#### 5.4.1. Mechanical Effects

The mechanical effects of blood flow in regions where arteries are exposed to high shear stress are protected from inflammation and atherosclerosis. By contrast, low-shear regions are susceptible and this effect has been shown to be due to the effect of Nrf2 in reducing activation of the endothelium at atherosusceptible sites [98].

#### 5.4.2. Pharmaceutical Drugs

The pharmaceutical tamoxifen, commonly prescribed to women following treatment for breast cancer, is an NQO1 inducer but its CD value is 30-fold lower than that for sulforaphane [99]. Nrf2 inducer activity may play some role in this drug's therapeutic profile in addition to its primary role as a selective estrogen receptor modulator (SERM) [99]. These comparative data may be clinically significant when considering the potential value of a drug or supplement with cytoprotective potential. A number of other pharmaceuticals pleiotropically activate Nrf2. The redox-modulating activity of the frequently prescribed statins and ACE inhibitors has been attributed to their Nrf2 inducer ability [100]. Similarly, gold salts, once the mainstay of treatment for rheumatoid arthritis, are Nrf2 inducers [101]. Indomethacin, now seldom used in reducing the symptoms of inflammatory joint diseases, has Nrf2-inducing properties, illustrating that nonsteroidal anti-inflammatory drugs (NSAIDs) exhibit properties other than their anti-inflammatory effects [102].

A relatively new pharmaceutical, Bardoxolone Methyl (BARD), was shown to enhance estimated glomerular filtration rate (eGFR) in patients with chronic kidney disease, a disease characterised by significant oxidative stress [103–105]. BARD is a synthetic analogue of oleanolic acid, a triterpenoid found extensively in edible plants [106], with broader cytoprotective properties attributed to Nrf2 induction [107]. The Phase 3 BEACON Trial [108] was halted in October 2012 following adverse events including 57 deaths out of 2185 participants in the BARD arm [105]. In comparing the inducer activity of BARD with that of SFN, a 2005 study comparing a range of triterpenoids showed that BARD was 230-fold more potent than SFN as a NQO1 inducer [109]. The adverse effects demonstrated by the synthetic triterpenoid analogue in the BEACON trial may be representative of a hormetic response at the upper end of a bifunctional dose response. By contrast, phytochemicals at the doses provided by foods are typically nontoxic [97].

#### 5.4.3. Exercise

Exercise is associated with an increased flux of glucose and oxygen through the mitochondria, a process which increases levels of ROS such as superoxide. An essential role for exercise-induced ROS formation in activating transcription factors and coactivators has been proposed [110]. Ristow et al. demonstrated that typical exercise-related changes in gene expression were almost completely abrogated by daily ingestion of supplements of vitamins C and E at doses of 1000 mg and 400 IU, respectively.

A review highlighted 23 studies showing that antioxidant supplementation interferes with exercise training-induced adaptations [111]. An emerging theme [112] supports the view that because Nrf2 is activated by a mild prooxidant signal, high doses of antioxidant supplements may blunt signals required to activate endogenous defenses [113, 114]. Ristow's assertion that antioxidant supplementation blocks many of the beneficial effects of exercise is supported by such evidence.

### 5.5. Other Actions of NQO1 Which Can Be Influenced by Sulforaphane

NQO1 exhibits broad substrate specificity extending well outside its better known role as a Phase II inducer; its other roles as described in the following section may contribute to its cytoprotective capacity. Its actions include the following: (1) it can protect against benzene-derived quinones such as benzo(a)pyrene, a carcinogen found commonly in petrochemical exhaust gases and in barbecued meats [115]; (2) NQO1 can reduce catechol estrogen quinones to catechol estrogens, a process associated with lowering breast cancer risk due to elevated estrogen metabolites [116]; (3) NQO1 can scavenge superoxide, albeit at a lower order of magnitude than does superoxide dismutase (SOD) [117], (4) NQO1 stabilises p53, the tumor suppressor gene [77]; and (5) NQO1 restores oxidized coenzyme Q10 (ubiquinone) and the tocopherols to their reduced forms [77].

Several NQO1 polymorphisms exist and these have been associated with risk of carcinogenesis. The C609T gene variant is one of very few common single nucleotide polymorphisms known to almost completely eliminate enzymatic activity; consequently, NQO1 is attracting considerable research attention given its multiple effects in cellular defenses [118].

### 5.6. Other Mechanisms: Animal Studies

Although a large volume of the published sulforaphane research is associated with its Nrf2 inducer potential, some studies point to other mechanisms. A recent study used broccoli sprout juice as the intervention material in stroke-prone spontaneously hypertensive rats to investigate possible effects on renal damage [119]. After 4 weeks, the animals were shown to have been largely protected against renal damage. Mechanistically, the effect was shown to be independent of systemic blood pressure but to parallel stimulation of the AMPK/SIRT1/PGC1a/PPARa/UCP2 axis. Whether this can be replicated in humans at practical doses has not yet been investigated.

## 6. The Issue of Bioavailability

### 6.1. Comparative Effects of Popular Phytochemical Supplements

Aside from wide variation in Nrf2 inducer capacity, a second barrier to clinical efficacy is bioavailability. When bioavailability is low, cell culture studies may significantly overestimate the intracellular concentration that ingestion of such a compound can achieve, being unlikely to demonstrate the expected clinical benefit indicated by the in vitro work [120, 121]. In considering the potential clinical efficacy of a phytochemical, the active compound and/or any of its active metabolites must reach the cells of the target organ(s) in appropriate concentration. Oral bioavailability of polyphenols is typically <10%, ranging between 2 and 20% [122], with many closer to 1%; cooking and processing significantly reduce polyphenol content [123]. By comparison, a pharmacokinetic animal study showed that sulforaphane was rapidly absorbed with its absolute bioavailability 82% [124].

Many phytochemical-containing supplements contain polyphenolic molecules such as curcumin (turmeric), catechins (green tea), resveratrol (grapes), ellagic acid (berries and pomegranate), and hydroxytyrosol and oleuropein (olives). Much of the evidence used to promote these supplements is from either in vitro or animal studies, with limited clinical evidence to support the assertions. Supplements of these phytochemicals frequently bear an “antioxidant” claim, even though the amount of polyphenol reaching the circulation or target cells is seldom adequate to alter redox status [7, 125]; gene expression studies have helped in quantifying likely systemic responses. Preclinical cell culture or animal studies may involve very high doses of an isolated polyphenol. Such doses are seldom clinically practical, considering average dietary intake of mixed polyphenols in food is approximately 1 gram per day of poorly bioavailable compounds [126].

**Figure 3:**
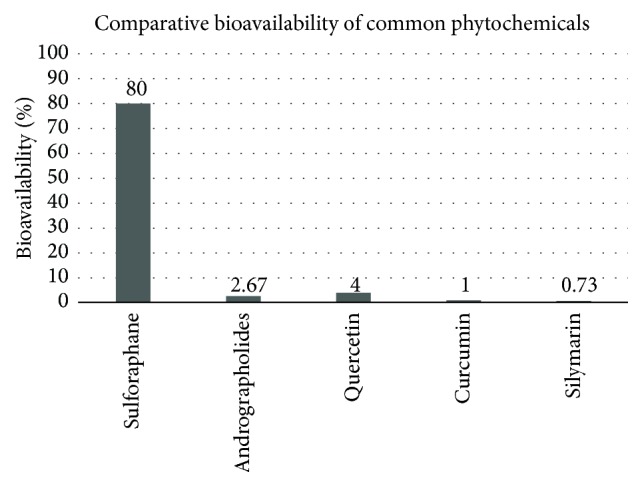
Comparative bioavailability of phytochemicals commonly used in supplements [90, 124, 127–129, 153].

Curcumin, resveratrol, and silybin are examples of popular polyphenol supplements for which preclinical findings cannot be readily extrapolated to the clinical environment.  Figure 3 compares the bioavailability of several polyphenols with that of sulforaphane (native curcumin at ~1% [127], resveratrol <1% [128], and silybin ~0.73% [129]). In each case, the systemic bioavailability compares the plasma concentration of an oral dose to an intravenous dose and is expressed as a percentage, where *F* is bioavailability [90]:

(1) $$\begin{array}{c} F^{\text{oral}}=\frac{\left({\text{AUC}}^{\text{oral}}/{\text{Dose}}^{\text{oral}}\right)}{\left({\text{AUC}}^{\text{i.v.}}/{\text{Dose}}^{\text{i.v.}}\right)}\times 100\%. \end{array}$$

The high intracellular concentrations of polyphenols required to replicate in vitro findings are difficult to achieve in humans with practical oral doses.

There is some evidence to suggest that the activity of some polyphenols may instead reside in their metabolites [130], so that small quantities absorbed intracellularly act as signaling molecules and may act synergistically with other biomolecules [131]. It is likely that any direct antioxidant effects occur only within the lumen of the gut [10, 132] and not systemically.

Quercetin naturally found in onions, watercress, tea, and other plants is a popular oral supplement typically promoted as an “antioxidant” or “anti-inflammatory” agent. Some studies suggest that quercetin may have anticancer potential [133] but other studies describe potential for risk [134], given that quercetin may exhibit prooxidant effects, especially in a GSH-depleted cellular environment [135].

Specifically, quercetin can exert an inhibitory effect on the metabolism of catechols via the catechol-O-methyltransferase enzyme (COMT) [136]. This may have implications in estrogen-related disorders where inappropriately metabolised estrogens can form DNA adducts [93]. Whether oral doses of quercetin have these effects in humans is not known but the issue has been flagged as “concerning” since readily available quercetin supplements represent up to 100 times the quantity typically available in a Western diet [137].

### 6.2. Curcumin

Curcumin is regarded as having in vitro anti-inflammatory activity by virtue of its ability to inhibit the transcription factor, NF-*κ*B [138]. In a study investigating inflammation in human tenocytes, high concentrations of 5–20 *μ*M were required to inhibit IL-1*β*-induced inflammation [139]. However, very high oral doses in humans (up to 8 g) yielded curcumin peak intracellular levels of only 0.5–2.0 *μ*M, clearly not attaining a concentration of the same order; commonly recommended supplemental doses of up to 180 mg were undetectable in plasma [140, 141]. Laboratory findings demonstrating an impressive and diverse array of cytoprotective effects for curcumin may not generally apply to practical oral doses in humans [142].

By contrast, there is evidence for an effect in gastrointestinal tissue, where transport occurs across a single enterocyte membrane [143, 144]. Patients with colorectal cancer were administered doses up to 3.6 g curcumin daily [145]. M_1_G, a marker of DNA damage, decreased 38% in the colorectal tissue, showing that a dose of 3.6 grams daily achieves pharmacologically efficacious levels in colonocytes but with negligible distribution outside the gut, confirming its poor systemic bioavailability.

When considering both CD value and bioavailability, native curcumin with bioavailability of ~1% would appear to be less clinically relevant than sulforaphane which shows both high inducer activity and high bioavailability. Even enhanced forms of curcumin with ~7-fold higher bioavailability still exhibit comparatively low bioavailability [146]. Investigating physiologically achievable doses of curcumin, Lao et al. administered from 500 to 12,000 mg of a curcumin powder; no curcumin was detected in any of 74 participants taking up to 8,000 mg; low serum levels in the ng/mL range were detected only for doses >8,000 mg, with doses below 4,000 mg barely detected [147]. Similarly, curcumin was not detected in normal liver or colorectal liver metastases in patients receiving 3.6 g/d for 1 week [145]. Howells et al. conclude that in vitro studies with curcumin in the high 10 *μ*mol/L range or below might have human physiological relevance but that its role as a chemopreventive agent may lie primarily within the gastrointestinal tract [141].

### 6.3. Resveratrol

Resveratrol achieved international acclaim after studies in mice and lower organisms indicated that it was responsible for a longevity effect [148]. Only mice injected with resveratrol from birth lived longer; those that started at middle age had no longevity benefit [149]. The benefit appeared due to enhanced expression of survival genes, a number of which are also expressed during caloric restriction [150].

The longevity effect has never been tested in humans, so an appropriate dose is not known nor even if a longevity benefit is likely [151, 152]. Although well absorbed, resveratrol displays low bioavailability; at least 70% of an oral 25 mg dose in human subjects was shown to appear as resveratrol metabolites in plasma, with most of the oral dose subsequently recovered in the urine [128]. Like curcumin, resveratrol is readily absorbed by enterocytes/colonocytes [153], showing potential benefit to intestinal tissues. A daily resveratrol dose of 3000 mg administered to overweight or obese men with nonalcoholic fatty liver disease (NAFLD) over 8 weeks did not significantly improve any of the features of NAFLD over placebo [154].

A review of 3650 publications on resveratrol concluded that the evidence is not sufficiently strong to justify a recommendation for resveratrol to humans beyond the dose which can be obtained from dietary sources, which is estimated to be ~4 mg daily for adults [155].

### 6.4. Silymarin

Silymarin, the major flavonoid complex in* Silybum marianum*, has a long history of traditional use in liver disorders [156]. Silymarin supplements claiming to target human liver detoxification mechanisms are readily available. Silibinin, the most bioactive of the complex, is insoluble in water and not lipophilic with low bioavailability of 0.73% in rats [129]. Its CD value ranks next below curcumin and third after sulforaphane. Where optimising Phase II detoxification is the desired outcome, there may be value in considering both CD values and bioavailability. Such evidence sheds considerable doubt on the likely efficacy of many such phytochemicals at doses typically found in commercially available supplements. Nevertheless, published trials show that silymarin exhibits hepatoprotective properties in humans, indicating that other mechanisms may be responsible [156, 157].

### 6.5. Sulforaphane

Sulforaphane's lipophilic nature and low molecular weight readily enable passive diffusion into cells [124]. It is rapidly absorbed, peaking in plasma as early as 1 hour after ingestion [158]. Predictably, dose-dependent pharmacokinetics in rats reveals that bioavailability decreases with increasing dose [124]. The doses corresponded to ~0.5 mg, 1.0 mg, and 5.0 mg/kg of pure sulforaphane which is relatively high for humans who typically consume* Brassica* vegetable and not pure sulforaphane. It is unlikely that humans through diet would ingest such high quantities of SFN. By calculation, MYR-active whole broccoli sprout supplement yielding 1% SFN could deliver 10 mg SFN per gram of powder, corresponding to ~12 grams of fresh broccoli sprouts (dried powder retains ~8% moisture). Administering 5.0 mg/kg of sulforaphane to a 70 kg human at the upper end of the animal dose range represents an intake of 350 mg or 35-fold the quantity that a human might reasonably ingest dietary fresh sprouts. Clearly, these quantities are not a practical means of providing a broccoli sprout supplement for human use.

### 6.6. Dose Considerations in Humans

An indication of what might be practically achievable with supplementation is illustrated by several human studies. Ye et al. showed that after a single 200 *μ*mol oral dose of sulforaphane both sulforaphane and its metabolites were detected in plasma and erythrocytes in just 15 minutes, peaking in all four subjects at ~2.00 *μ*M after 1 hour and declining with first-order kinetics, with a mean half-life of 1.77 ± 0.13 hours [159]. To investigate effects in systemic tissue, Cornblatt et al. showed that, one hour after a single 200 *μ*mol oral dose of sulforaphane administered to 8 women, metabolites were detected in resected left and right breast tissue at concentrations of 1.45 ± 1.12 and 2.00 ± 1.95 pmol/mg tissue, respectively. This proof-of-principle study observed a significant induction of NQO1 enzymatic activity in the same tissue [158]. In another example, a dose escalation placebo-controlled study investigated Phase II gene expression in human airways mucosa, showing that a 200-gram broccoli sprout homogenate delivering 102 *μ*mol of sulforaphane increased NQO1 mRNA expression by almost 200% [160].

Given that oral doses appear to be capable of increasing NQO1, we consider whether it may be possible that a sulforaphane-yielding broccoli sprout powder might deliver a plasma concentration of ~2.00 *μ*M. By calculation, a 1% powder yields 56.4 *μ*mol sulforaphane per gram. Ye et al. showed that a single 200 *μ*mol dose resulted in a peak plasma concentration of ~2.0 *μ*M after 1 hour. As Ye et al. [159] had shown that a 200 *μ*mol oral dose had resulted in a plasma concentration of ~2.0 *μ*M and Riedl et al. [160] had shown that 102 *μ*mol had increased NQO1 mRNA expression by ~200%; these orders of magnitude could be achievable with a sulforaphane-yielding broccoli sprout powder. Theoretically and by calculation, an individual could consume around 2 grams of a 1% sulforaphane-yielding broccoli sprout powder to achieve what Riedl et al. achieved with 200-gram broccoli homogenate and 4 grams to achieve what Ye et al. achieved with a single 200 *μ*mol dose.

## 7. Factors Governing Sulforaphane Yield

### 7.1. The Role of Myrosinase

Glucosinolates as* Brassica*-derived precursor compounds are converted to their bioactive forms only under the action of MYR because GRN has no inherent bioactivity. Investigating the metabolic fate of ingested broccoli phytochemicals, Shapiro et al. showed that MYR-inactivated broccoli resulted in 10–20% lower conversion to ITCs. When the colonic microfloras were reduced, recovery of ITCs in a MYR-free environment was negligible. It may be inferred that MYR is essential for sulforaphane synthesis and that the colonic microflora may exhibit MYR-like activity.

The colonic microfloras appear to be capable of limited MYR activity, with conversion to the bioactive ITC varying from 1% to 40% of the dose [161]. Several genera of human microflora such as* Bifidobacterium*,* Lactobacillus*, and* Bacteroides* have been reported to possess MYR-like activity [162] but with wide variability in their population; the ability to hydrolyse glucosinolates cannot be reliably estimated. So unpredictable is this factor that a large clinical trial using MYR-inactive broccoli sprout extract could not achieve statistical significance [163]. Many available broccoli sprout supplements are MYR-inactive extracts which claim their clinical benefit is due to the alleged conversion to sulforaphane by the colonic microflora. Neither consumers nor clinicians have any way of knowing if an individual harbors MYR-active microflora.

### 7.2. The Nitrile Factor

Among crucifers, broccoli contains significant amounts of epithiospecifier protein (ESP), a noncatalytic inhibitor of MYR activity [164]. ESP produces inactive sulforaphane nitrile. Under certain conditions, the nitrile pathway is favoured, with the hydrolysis product constituting as much as 75% nitrile. The colonic microfloras also support nitrile formation, thereby further limiting the potential of MYR-inactive supplement [165]. ESP deactivation can significantly enhance sulforaphane yield, illustrating that broccoli and broccoli sprout products cannot be meaningfully evaluated on the basis of their GRN content alone. It is likely that clinical trials using either fresh or powdered broccoli sprouts may give conflicting results when the presence or absence of nitrile has not been considered. The presence of ESP means that assayed measurement of the sulforaphane yield is critical in order to estimate the real efficacy of a broccoli sprout powder intended for a supplement; measurement only of GRN and MYR does not allow for the effect of ESP on enzyme activity.

## 8. Clinical Implications

### 8.1. Cruciferous Vegetable Consumption

The presence of unquantified amounts of ESP in raw broccoli has clinical implications; as a salad vegetable, raw broccoli may not be an efficient means of obtaining the benefits conferred by sulforaphane. Similarly, cooking has been shown to destroy the enzyme in as little as 3 minutes of steaming [166]. Five minutes of microwave cooking resulted in 74% loss of glucosinolates from broccoli florets with high-pressure cooking and boiling leading, respectively, to 33% and 55% losses [83]. Even consumers and clinicians conscious of the importance of cruciferous vegetables in the diet may be unaware that open-air storage of broccoli as occurs during transport and in retail environments may lose 55% of its glucosinolates after 3 days and storage in plastic bags at 22°C may result in similar losses over 7 days [83].

Also of significance is the fact that broccoli cultivars for vegetable production are not selected on the basis of their sulforaphane yield. It is possible that the cultivars available to consumers are not good sources of cruciferous bioactives. Until Food Law allows appropriate health claims to be associated with cruciferous vegetables, there is no incentive for growers to select higher yielding cultivars. In short, neither a clinician nor a consumer has the information needed to make an appropriate choice.

### 8.2. Supplements Derived from Cruciferous Vegetables

Similarly, it is not generally known if a producer of broccoli sprout powder as a supplement ingredient has deactivated the ESP. If two supplements contain high levels of GRN but one has had the ESP deactivated, the comparative sulforaphane yield from these broccoli sprout powders may be markedly different. Ideally, a sulforaphane-yielding supplement would be characterised on the basis of the various determining factors: the presence of quantifiable GRN and active MYR together with the inhibitory ESP.

These concerns are reflected in a recent study which compared a commercially available supplement labelled as containing 30 mg “sulforaphane glucosinolate” per dose with a quantity of fresh sprouts containing the same amount of GRN [167]. The study showed that consumption of MYR-devoid broccoli supplement when compared with broccoli sprouts produced 7-fold lower plasma concentrations of the bioactive ITC metabolites in the subjects. Clarke et al. concluded that these findings have implications for people who consume the recommended dose of such MYR-inactive broccoli supplement believing they are obtaining equivalent doses of ITCs. This is significant in that the available broccoli sprout supplements are dominated by the MYR-inactive “extracts,” even though MYR-active whole broccoli sprout supplements do exist.

There is a further strong case for a whole food broccoli sprout supplement on the grounds that although GRN is the primary glucosinolate found in broccoli and broccoli sprouts, it is not the only one; erucin and iberin comprise most of the remaining 25% of the glucosinolate content of broccoli. Recently, it was found that erucin and sulforaphane are interconvertible, so that the clinical effects are likely to be due to the combined effects of all the glucosinolate hydrolysis products [167].

## 9. Standardisation

To compound the difficulties associated with determining the clinical potential of a sulforaphane-yielding supplement, variations in nomenclature add to the problem. The term “*sulforaphane glucosinolate*” which has recently appeared in the scientific literature is now associated with and specified for commercially available MYR-inactive extracts derived from broccoli seed or sprout extracts [167, 168]. Since “sulforaphane glucosinolate” describes only the quantity of “glucoraphanin,” this nomenclature could erroneously lead both clinicians and consumers to believe that the material will deliver sulforaphane when consumed.

### 9.1. Commercial Assay Protocols

Various methods to describe a sulforaphane supplement are commonly used in industry. To evaluate and compare different broccoli sprout powders intended as supplements or for use in clinical trials, assay methodologies must be standardised. There are several common practices for reporting the sulforaphane derived from a broccoli sprout sample but because the assay protocol is almost never specified for a commercial product there is no way to reliably compare these values from one product to another.

### 9.2. Sulforaphane Potential

Sulforaphane potential is a calculated value by measuring GRN and then assuming 100% conversion to sulforaphane, whether or not MYR has been retained after processing. Based on relative molecular weights, the measured amount of GRN is multiplied by 0.406 to arrive at a sulforaphane potential. No provision is made for the presence or absence of either MYR or ESP. Where ESP has not been fully deactivated, calculating sulforaphane potential will overestimate the amount of sulforaphane that could be produced on ingestion. Broccoli sprout powdered ingredients or supplements which claim sulforaphane potential and for which the ESP has not been deactivated may yield limited sulforaphane.

### 9.3. Sulforaphane Yield with Addition of Exogenous MYR

By adding enough exogenous MYR to ensure full conversion of GRN to sulforaphane, this method overcomes the possibility that the starting material may contain only GRN and may be completely or partially MYR-inactive. The assay results may not specify that exogenous MYR was added, so that the reader may incorrectly conclude that the material will yield sulforaphane on ingestion.

### 9.4. Sulforaphane Yield due to Endogenous MYR

This method more closely resembles the in vivo situation after ingestion of the supplement, in that conversion to sulforaphane is entirely dependent on the quantities of ESP and MYR retained after processing. It may provide a lower sulforaphane value when compared with the other methods, even though it may be the method which most reliably approximates sulforaphane's metabolic fate in human physiology.

The same supplement assayed by each of these procedures is likely to produce quite different results and, more importantly, only supplements which have retained MYR activity are likely to demonstrate in vivo effects. Methods which assess sulforaphane's inducer capacity in cell culture may more reliably evaluate the clinical potential of a supplement or enable comparison of supplements. PCR array and pathway analysis studies provide gene expression data which is another closer step to establishing the clinical effects of a supplement [169].

## 10. Conclusion

The evolving science of nutrigenomics is in many ways legitimizing the important role of plant foods in human health, not just as sources of nutrients but as a huge library of phytochemicals capable of interacting with intracellular biomolecules to influence gene expression. Of the many thousands of phytochemicals in the food supply, sulforaphane exhibits properties which may make it an ideal cytoprotective biomolecule, deliverable in practical doses as a whole food supplement. Commercial attempts to produce sulforaphane-releasing supplements have resulted mostly in forms with little or no bioactivity, typically seed or sprout extracts. The ideal sulforaphane-releasing supplement retains both its glucoraphanin precursor and its myrosinase enzyme in the form of a whole broccoli sprout ingredient with nothing but water removed. When compared with other phytochemicals widely used in dietary supplements, sulforaphane is significantly more bioavailable than polyphenols such as curcumin, resveratrol, and silymarin. It is also significantly more able to induce NQO1, a Phase II enzyme essential in the metabolism of a number of exogenous toxins, oxidized nutrients, and endogenous metabolites. Such comparative findings call into question the clinical efficacy of many of the supplements popular among consumers. Alleged benefits of such supplements appear to require much higher intracellular concentrations than can be achieved with reasonable oral intake.

Initial attempts to produce high-potency pharmaceutical Nrf2 inducers have so far been unsuccessful. Given the prevalence of diet-related disease and the evidence that many consumers have accepted a role for complementary medicines in their personal health management, appropriately validated sulforaphane-releasing supplements may provide another avenue for supporting human health. Such supplements will need to demonstrate sufficient nutrigenomic potential that they can modify key biochemical and physiological risk factors for disease.

## Supplementary Material

The following supplementary material illustrates sulforaphane's chemical structure, synthesis and interactivity, together with its effects on gene expression in key pathways.
Table 1: Major Products of Nrf2 Target Genes
Figure 1: Molecular structure of sulforaphane
Figure 2: Sulforaphane synthesis via myrosinase enzyme
Figure 3: Epithiospecifier protein, an inhibitor of myrosinase enzyme
