# Supplementary material for: Sulforaphane and Other Nutrigenomic Nrf2 Activators: Can the Clinician's Expectation Be Matched by the Reality?
Source: Oxid Med Cell Longev. 2016 Jan 6;2016:7857186. doi: 10.1155/2016/7857186 (PMC4736808; doi:10.1155/2016/7857186)
Supplement: Supplementary file 1 — The following supplementary material illustrates sulforaphane's chemical structure, synthesis and interactivity, together with its effects on gene expression in key pathways. Table 1: Major Products of Nrf2 Target Genes Figure 1: Molecular structure of sulforaphane Figure 2: Sulforaphane synthesis via myrosinase enzyme Figure 3: Epithiospecifier protein, an inhibitor of myrosinase enzyme [file 7857186.f1.docx]

**Sulforaphane and other nutrigenomic Nrf2 Activators: can the clinician’s expectation be matched by the reality?**

**SUPPLEMENTARY DATA**

**Table 1 Major Products of Nrf2 Target Genes**

| PRODUCTS OF Nrf2-TARGET GENES | ROLE IN CYTOPROTECTION |
| --- | --- |
| Glutathione (non-enzyme)  (GSH) | Abundant intracellular sulfur-containing direct antioxidant – predominant intracellular thiol.[[1](#_ENREF_1)] Essential in function of Glutathione peroxidase and GST for redox balance and detoxification.[[1](#_ENREF_1)] |
| Haemoxygenase-1  (HO-1) | Redox-regulating, broad protection against oxidative stress.[[2](#_ENREF_2)] Metabolises haem, also producing bilirubin which scavenges peroxyl radicals. Anti-inflammatory and immune-modulating properties.[[3](#_ENREF_3)] |
| Thioredoxin (Trx) (non-enzyme) | Ubiquitous intracellular sulfur-rich protein. Singlet oxygen quencher and hydroxyl radical scavenger.[[4](#_ENREF_4)] |
| Thioredoxin reductase  (TrxR) | An oxido-reductase which regenerates Trx and GSH.[[5](#_ENREF_5)] |
| Glutathione-S-transferase  (GST) | A Phase II detoxifying enzyme with broad spectrum of activity, depending on subclass.[[6](#_ENREF_6)] |
| Quinone reductase  NAD(P)H:Quinone oxido-reductase (NQO1) | A multifunctional redox-regulating and detoxifying enzyme, including protection against oestrogen quinone metabolites.[[7](#_ENREF_7)] Directly scavenges superoxide but less efficiently than SOD.[[8](#_ENREF_8)] Stabilises the p53 tumor suppressor protein,[[9](#_ENREF_9)] especially under exposure from γ-irradiation or other oxidative stress. Protective against dopamine cytotoxicity where SOD and Catalase were not.[[10](#_ENREF_10)] |
| Ferritin | Binding of free iron to prevent its reaction with superoxide to produce hydroxyl radical.[[11](#_ENREF_11)] |
| Metallothionein | Removal of heavy metals such as mercury and cadmium.[[12](#_ENREF_12)] |
| Peroxisome proliferator-activated receptor  (PPAR-γ) | Regulator of adipogenesis and central integrator of glucose metabolism, energy homeostasis and skeletal metabolism.[[13](#_ENREF_13)] |
| Nuclear factor erythroid 2-related factor 2  (Nrf2) | Nrf2 induces its own synthesis.[[14](#_ENREF_14)] |
| NADPH regenerative enzymes | Restores reducing equivalents and reduces oxidized GSH to its reduced form.[[9](#_ENREF_9)] |

**FIGURES**

**
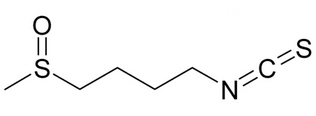
MOLECULAR STRUCTURE OF SULFORAPHANE**

Figure 1 Sulforaphane: molecular structure – a low Molecular Weight (MW = 177.29) lipophilic molecule


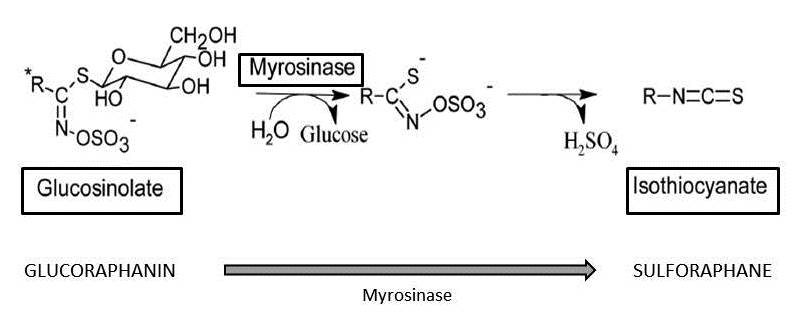
**SULFORAPHANE SYNTHESIS VIA MYROSINASE ENZYME**

Figure 2 How isothiocyanates such as sulforaphane are enzymatically-derived from their precursor glucosinolates such as glucoraphanin.

**EPITHIOSPECIFIER PROTEIN, AN INHIBITOR OF MYROSINASE ENZYME**


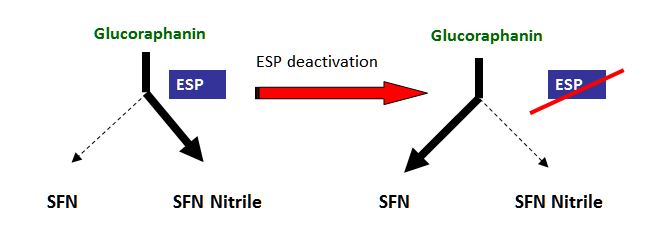


**Figure 3** The presence of Epithiospecifier Protein (ESP) prevents complete conversion of glucoraphanin to sulforaphane. Instead, part of the glucoraphanin is converted to inactive sulforaphane nitrile. As much as 75% of the product of myrosinase activity on glucoraphanin can be sulforaphane nitrile.

**REFERENCES for Table 1 Data:**

1. Yuan, L.; Kaplowitz, N., Glutathione in liver diseases and hepatotoxicity. *Mol Aspects Med* **2009,** *30* (1-2), 29-41.

2. Morse, D.; Choi, A. M., Heme oxygenase-1: from bench to bedside. *Am J Respir Crit Care Med* **2005,** *172* (6), 660-70.

3. Blancou, P.; Tardif, V.; Simon, T.; Remy, S.; Carreno, L.; Kalergis, A.; Anegon, I., Immunoregulatory properties of heme oxygenase-1. *Methods Mol Biol* **2011,** *677*, 247-68.

4. Hu, Y.; Urig, S.; Koncarevic, S.; Wu, X.; Fischer, M.; Rahlfs, S.; Mersch-Sundermann, V.; Becker, K., Glutathione- and thioredoxin-related enzymes are modulated by sulfur-containing chemopreventive agents. *Biol Chem* **2007,** *388* (10), 1069-81.

5. Nishinaka, Y.; Nakamura, H.; Masutani, H.; Yodoi, J., Redox control of cellular function by thioredoxin; a new therapeutic direction in host defence. *Arch Immunol Ther Exp (Warsz)* **2001,** *49* (4), 285-92.

6. Fahey, J. W.; Talalay, P., Antioxidant functions of sulforaphane: a potent inducer of Phase II detoxication enzymes. *Food Chem Toxicol* **1999,** *37* (9-10), 973-9.

7. Cavalieri, E.; Chakravarti, D.; Guttenplan, J.; Hart, E.; Ingle, J.; Jankowiak, R.; Muti, P.; Rogan, E.; Russo, J.; Santen, R.; Sutter, T., Catechol estrogen quinones as initiators of breast and other human cancers: implications for biomarkers of susceptibility and cancer prevention. *Biochimica et biophysica acta* **2006,** *1766* (1), 63-78.

8. Siegel, D.; Gustafson, D. L.; Dehn, D. L.; Han, J. Y.; Boonchoong, P.; Berliner, L. J.; Ross, D., NAD(P)H:quinone oxidoreductase 1: role as a superoxide scavenger. *Mol Pharmacol* **2004,** *65* (5), 1238-47.

9. Dinkova-Kostova, A. T.; Talalay, P., NAD(P)H:quinone acceptor oxidoreductase 1 (NQO1), a multifunctional antioxidant enzyme and exceptionally versatile cytoprotector. *Arch Biochem Biophys* **2010,** *501* (1), 116-23.

10. Zafar, K. S.; Inayat-Hussain, S. H.; Siegel, D.; Bao, A.; Shieh, B.; Ross, D., Overexpression of NQO1 protects human SK-N-MC neuroblastoma cells against dopamine-induced cell death. *Toxicol Lett* **2006,** *166* (3), 261-7.

11. Emerit, J.; Beaumont, C.; Trivin, F., Iron metabolism, free radicals, and oxidative injury. *Biomed Pharmacother* **2001,** *55* (6), 333-9.

12. Yeh, C. T.; Yen, G. C., Effect of sulforaphane on metallothionein expression and induction of apoptosis in human hepatoma HepG2 cells. *Carcinogenesis* **2005,** *26* (12), 2138-48.

13. Astapova, O.; Leff, T., Adiponectin and PPARgamma: cooperative and interdependent actions of two key regulators of metabolism. *Vitam Horm* **2012,** *90*, 143-62.

14. Kwak, M. K.; Itoh, K.; Yamamoto, M.; Kensler, T. W., Enhanced expression of the transcription factor Nrf2 by cancer chemopreventive agents: role of antioxidant response element-like sequences in the nrf2 promoter. *Mol Cell Biol* **2002,** *22* (9), 2883-92.
